# Design and Rationale for the RETAIN Trial of Non‐invasive Brain Stimulation of the Default Mode Network for Mild to Moderate Alzheimer’s Disease

**DOI:** 10.1002/alz70859_107435

**Published:** 2025-12-26

**Authors:** Lisa Fosdick, Giacomo Koch

**Affiliations:** ^1^ Sinaptica Therapeutics, Cambridge, MA USA; ^2^ IRCCS Santa Lucia Foundation, Rome Italy

## Abstract

**Background:**

Altered cortical plasticity is considered a central neurophysiological feature of AD and non‐invasive brain stimulation may represent a valid tool to restore altered cortical plasticity by promoting synaptic activity. Prior clinical trials have demonstrated the safety and feasibility of neuromodulation of the default mode network (nDMN) via the precuneus for the treatment of mild to moderate AD. These previous clinical trials have provided guidance for the design of the RETAIN trial.

**Method:**

RETAIN is designed as a prospective, multi‐center, double‐blind randomized controlled trial of nDMN in patients with mild to moderate AD. Key inclusion criteria include a clinical diagnosis of stage 4 or 5 AD with a positive biomarker profile, global CDR 0.5, 1.0 or 2.0 and memory decline over the prior 6 months. Key exclusions include significant neurological and cerebrovascular diseases, current memantine use, inability to tolerate the TMS‐EEG procedure and lack of ability to elucidate a treatment evoked potential during screening. Minimum sample size is 120 in a seamless adaptive P2/P3 study design including interim analyses allowing sample size adjustment. Treatment is a 10‐day initiation period followed by 22 weekly treatment sessions delivered via the Sinaptistim system. Personalized selection of treatment target and intensity will be obtained through an integrated software program incorporating the neurophysiological data obtained through TMS‐EEG. All participants will follow the same treatment schedule with two‐thirds receiving active treatment and one‐third receiving treatment via a sham coil.

**Result:**

The study will evaluate efficacy using changes in cognition and function via standard neuropsychological assessments including ADAS‐Cog_13_, ADCS‐ADL, and CDR. Magnetic resonance imaging (MRI) and fMRI will be obtained via an imaging core laboratory and used for key analyses. Plasma biomarkers of neurodegeneration and synaptic activity will be obtained on all participants.

**Conclusion:**

The RETAIN trial is designed to evaluate the efficacy and safety of nDMN in patients with mild to moderate AD. Several sub‐studies and exploratory analyses are also planned to further elucidate the mechanism of action of this treatment modality. The RETAIN trial will be conducted as the pivotal trial to support a De Novo classification request to the FDA.

